# Diagnostic Applications of Cone-Beam CT for Periodontal Diseases

**DOI:** 10.1155/2014/865079

**Published:** 2014-04-03

**Authors:** Yousef A. AlJehani

**Affiliations:** Dental Health Department, College of Applied Medical Sciences, King Saud University, P.O. Box 10219, Riyadh 11433, Saudi Arabia

## Abstract

*Objectives*. This paper aims to review the diagnostic application of cone beam computed tomography (CBCT) in the field of periodontology. *Data*. Original articles that reported on the use of CBCT for periodontal disease diagnosis were included. *Sources*. MEDLINE (1990 to January 2014), PubMed (using medical subject headings), and Google Scholar were searched using the following terms in different combinations: “CBCT,” “volumetric CT,” “periodontal disease ,” and “periodontitis.” This was supplemented by hand-searching in peer-reviewed journals and cross-referenced with the articles accessed. *Conclusions*. Bony defects, caters, and furcation involvements seem to be better depicted on CBCT, whereas bone quality and periodontal ligament space scored better on conventional intraoral radiography. CBCT does not offer a significant advantage over conventional radiography for assessing the periodontal bone levels.

## 1. Introduction

Periodontal disease is a chronic bacterial infection that affects the gingiva and bone supporting the teeth [1]. Treatment of patients with advanced periodontal diseases requires not only extensive clinical recording but also radiological examination [2]. Radiography provides vital information on the amount and type of damage to the alveolar bone [3]. The current diagnostic approaches including clinical probing and intraoral radiography have shown several limitations in their reliability [4–6].

Intraoral radiography is the most commonly used imaging technique for the diagnosis of periodontal bone defects. However, intraoral radiography provides only a 2-dimensional (2D) view of 3-dimensional (3D) structures which can lead to underestimation of bone loss and errors in identifying reliable anatomical reference points [4, 5, 7]. Three-dimensional (3D) diagnostic imaging of the jaws has been of interest from the introduction of computerized tomography (CT) as a clinical tool. However, due to the factors like high cost and high radiation dosage, use of this technology in dentistry has been limited.

Cone-beam computed tomography (CBCT) is a relatively new imaging modality and with the introduction of dedicated dentomaxillofacial CBCT scanners in the late 1990s [8, 9], there has been an explosion of interest in these devices in the field dentistry. It has the obvious advantage of relatively low-cost and low-dose [10].

CBCT differs from CT in that it uses a single X-ray source that produces a cone beam of radiation (rather than a fan beam, as with CT) [11]. CBCT uses a single, relatively inexpensive, flat-panel or image intensifier radiation detector. CBCT imaging is performed using a rotating platform to which the X-ray source and detector are fixed. As the X-ray source and detector rotate around the object, it produces multiple, sequential, and planar images that are mathematically reconstructed into a volumetric dataset. A single rotational sequence would capture enough data for volumetric image construction. The entire scanning of the target region is performed in a single rotation thereby significantly reducing the radiation exposure. Further, the exposure is reduced by 50% (0.0037 mGy) if a 180° scan is performed instead of 360° [12]. In comparison, the radiation exposure in a digital panoramic radiograph is around 0.0063 mGy and around 0.0012 mGy in a periapical radiograph [13]. It has been reported that for an intraoral status of the entire dentition an effective dose ranging from 33 to 84 Sv is required [14].

CBCT as a diagnostic tool is widely used in dentoalveolar surgeries [15–17], implantology [18, 19], general/specialized dentistry (orthodontics, endodontics, periodontics, and forensic dentistry) [20–23], and otolaryngology [24]. The currently available CBCT devices are capable of providing panoramic and cephalometric images. Additionally, the low footprint of these devices makes it suitable for dental office placement, therefore producing high quality images of specific regions of interest.

Few studies have appraised the role of CBCT in the diagnosis of periodontal diseases. This review aims to assess the diagnostic application of CBCT in the field of periodontology.

*Search Strategy*. MEDLINE (1990 to January 2014), PubMed (using medical subject headings), and Google Scholar were searched using the following terms in different combinations: “CBCT,” “volumetric CT,” “periodontal disease,” and “periodontitis.” This was supplemented by hand-searching in peer-reviewed journals and cross-referenced with the articles accessed.

## 2. CBCT in Periodontology

The success of periodontal therapy depends on many factors. One of the most important factors is an accurate image of the morphology of periodontal bone destruction to plan the treatment plan [25]. Radiographs are necessary to determine the extent and severity of the periodontal lesions [5, 6]. To view the periodontal structures, intra- and extraoral imaging modalities are available. The more commonly used method is the intraoral radiographs which provide a two-dimensional view. The extraoral panoramic radiographs are also used, especially to view larger areas. The major disadvantage of this method is the distortion of the images and the blurring of anatomical structures. Also, three-dimensional information is represented in a two-dimensional plane, thus losing essential diagnostic details [7]. When compared to periodontal probing and 2D intraoral radiography, 3D CBCT scanning was found to be more effective in assessing periodontal structures [26]. CBCT had better potential of detecting periodontal bone defects in all directions compared with periapical radiographs and were as reliable as radiographs for interproximal areas [26]. Misch et al. [26] reported that CBCT is as accurate as direct measurements using a periodontal probe and as reliable as intraoral radiographs for interproximal areas. Also, since buccal and lingual defects could not be diagnosed with intraoral radiography, CBCT could be considered a superior technique. Considering the various benefits, CBCT is currently being considered as a superior diagnostic tool for applications in periodontology [27].

### 2.1. CBCT in Diagnosing Furcations, Caters, and Bony Defects

Detection of bone defects or furcation involvement poses significant challenges for the practitioner [28]. Earlier studies have shown that computed tomography (CT) assessment of periodontal bone height and intrabony defects is reasonably accurate and precise [29–32]. However, the higher radiation exposure could not always be adequately justified.

Noujeim et al. [33] created periodontal lesions of different depths in dried human hemimandibles and analyzed them using intraoral radiography and CBCT. They found that CBCT was more accurate in detecting the defects than the conventional radiograph [34]. Similarly, other studies have reported higher precision in diagnosing periodontal defects, particularly, in the orovestibular orientation using CBCT compared with conventional radiograph [25, 34].

Stavropoulos and Wenzel [35] evaluated the accuracy of CBCT scanning with intraoral periapical radiography for the detection of periapical bone defects. CBCT was found to have better sensitivity compared to intraoral radiography. Various in vitro studies have stated that CBCT is effective in identifying and measuring artificially created defects on samples [26, 31].

Leung et al. [36] evaluated the accuracy and reliability of CBCT in the diagnosis of naturally occurring bone defects by comparing the difference between the CBCT measurements and measurements made directly on the skulls. They reported that CBCT measurements were not as accurate as direct measurements on skulls. A certain discrepancy between direct measurements and estimated measurements on radiographs has to be considered as clinically acceptable [34]. Further, with the development of advanced equipment and software the diagnostic ability of CBCT has improved. A recent study reported on an improved quantification of periodontal bone defects based on CBCT datasets using a new software [20]. These studies provide promising data promoting the use of CBCT for the detection of periodontal bony defects.

Vandenberghe et al. [34] studied thirty periodontal bone defects of 2 adult human skulls using intraoral digital radiography and CBCT. Periodontal bone levels and defects on both imaging modalities were assessed and compared to the gold standard. The study concluded that the intraoral radiography was significantly better for contrast, bone quality, and delineation of lamina dura, but CBCT was superior for assessing crater defects and furcation involvements.

### 2.2. CBCT in Measuring Periodontal Bone Levels

Sufficient alveolar bone volume and favorable architecture of the alveolar ridge are essential to obtain ideal functional and esthetic prosthetic reconstruction [37]. Studies of the extent of vertical alveolar bone defects from radiographs and from exploratory surgery have also indicated a good agreement between the radiographic and the clinical findings [38, 39]. Persson et al. [39] reported that conventional radiographic images provided a better resolution of the bone levels than what can be achieved from computer screen images.

Infrabony defects are the main cause of tooth loosening and loss and are often overlooked in researchers pertaining to the validation of radiographic modalities for periodontal diagnosis [2, 40]. CBCT provides high resolution images that can be used to gather diagnostic and quantitative information on periodontal bone health. The 3D images are ideal for evaluating the infrabony defects and assessing the treatment outcomes. Mol and Balasundaram [27] compared the image quality between CBCT and conventional radiography in the assessment of alveolar bone levels. They found that CBCT provided slightly better diagnostic and quantitative information on periodontal bone levels in three dimensions than conventional radiography. They found that the accuracy in the anterior aspect of the jaws is limited in both imaging techniques, obtained with traditional means.

Alternatively, numerous studies have reported that the CBCT images provided comparable measurements of periodontal bone levels and defects as intraoral radiography [26, 41]. Vandenberghe et al. [34] reported that CBCT images demonstrated more potential in the morphological description of periodontal bone defects, while the digital radiography provided more bone details.

### 2.3. CBCT in the Visualization of Periodontal Ligament Space

A break in the continuity of lamina dura and a wedge-shaped radiolucent area at the mesial or distal aspect of the periodontal ligament space are one of the earliest signs of periodontal disease [42]. However, it is clear that this does not occur until sometime after the loss of soft tissue attachment [43]. Therefore, only a sensitive imaging technique would be able to detect the earliest changes in the periodontal ligament space. The conventional intraoral radiographs have some significant disadvantages including the overlap of anatomical structures due to the positioning of the X-ray tube. Also, there could be errors related to the chemical processing and patient positioning [44]. Considering the potential advantages of using CBCT for assessing the periodontal structures, only very few studies have used it for visualization of the periodontal ligament space [45, 46]. In terms of image quality, the CBCT scans were found to be superior to the CT scans with particular reference to the periodontal ligament space [32]. Various studies have stated that conventional radiographs are better than CBCT in assessing the periodontal ligament space [34, 47]. Özmeric et al. [47] created a phantom model with artificial periodontal ligament space to compare between CBCT and conventional radiographs. They found that the CBCT was inferior to conventional radiographs in terms of the clarity of the artificial periodontal ligament space. However, conflicting views were reported in an in vitro study that found CBCT to be better than conventional radiography in visualizing the periodontal ligament space [48]. A phantom mimicking variable periodontal ligament spaces was radiographed using CBCT and intraoral radiographs [48]. This study found that CBCT provided better visualization of simulated periodontal ligament space in this phantom.

### 2.4. Other Periodontal Applications of CBCT

Even within the field of periodontology, CBCT has found numerous applications. CBCT has been widely used in the detection of periapical pathology. Various studies have reported on the effectiveness of using CBCT for the diagnosis of periapical pathology [49–51]. The literature shows that CBCT images are superior for the detection of apical periodontitis than conventional radiographs [49–51]. Apical periodontitis is one of the most common endodontic diseases, and it is considered to be the primary indication for root canal treatment and a sequela of inadequate or failing treatment [52]. CBCT has found application even in epidemiological surveys. A recent study [53] used CBCT images from a database to determine the prevalence of apical periodontitis. Dutta et al. [23] investigated the prevalence of periradicular periodontitis using CBCT scans in a retrospective cross-sectional epidemiological study in a Scottish subpopulation. CBCT is a radiological technique that has been more successful in detecting periradicular changes than conventional radiography [54]. In a recent case-report, CBCT was used in the diagnosis of a palatogingival groove [55]. Another recent study evaluated bone resorption at the extraction sites of a group of patients under orthodontic treatment using CBCT to evaluate the periodontal and bone support loss after tooth extraction [56]. CBCT was preferred due to its higher precision in detecting bone changes.

## 3. Conclusion

As the radiation dosage of CBCT is substantially higher than that of other routine dental imaging techniques, appropriate patient selection criteria must be adopted [57]. Also, the influence of the technical conditions on the image quality is relatively higher for CBCT. CBCT has the potential to gather accurate diagnostic and quantitative information about periodontal bone condition. Bony defects, caters, and furcation involvements seem to be better depicted on CBCT, whereas bone quality and periodontal ligament space scored better on conventional intraoral radiography. CBCT does not offer a significant advantage over conventional radiography for assessing the periodontal bone levels. Decision pertaining to the use of CBCT in the field of periodontology should be taken after careful consideration of its advantages, limitations, and risks.
